# Acupuncture Modulates Resting State Hippocampal Functional Connectivity in Alzheimer Disease

**DOI:** 10.1371/journal.pone.0091160

**Published:** 2014-03-06

**Authors:** Zhiqun Wang, Peipeng Liang, Zhilian Zhao, Ying Han, Haiqing Song, Jianyang Xu, Jie Lu, Kuncheng Li

**Affiliations:** 1 Department of Radiology, Xuanwu Hospital of Capital Medical University, Beijing, China; 2 Department of Neurology, Xuanwu Hospital of Capital Medical University, Beijing, China; 3 General Hospital of Chinese People’s Armed Police Forces, Beijing, China; 4 Key Laboratory for Neurodegenerative Diseases, Ministry of Education, Beijing, China; 5 Beijing Key Laboratory of Magnetic Resonance Imaging and Brain Informatics, Beijing, China; Imperial College London, Chelsea & Westminster Hospital, United Kingdom

## Abstract

Our objective is to clarify the effects of acupuncture on hippocampal connectivity in patients with Alzheimer disease (AD) using functional magnetic resonance imaging (fMRI). Twenty-eight right-handed subjects (14 AD patients and 14 healthy elders) participated in this study. Clinical and neuropsychological examinations were performed on all subjects. MRI was performed using a SIEMENS verio 3-Tesla scanner. The fMRI study used a single block experimental design. We first acquired baseline resting state data during the initial 3 minutes and then performed acupuncture stimulation on the Tai chong and He gu acupoints for 3 minutes. Last, we acquired fMRI data for another 10 minutes after the needle was withdrawn. The preprocessing and data analysis were performed using statistical parametric mapping (SPM5) software. Two-sample t-tests were performed using data from the two groups in different states. We found that during the resting state, several frontal and temporal regions showed decreased hippocampal connectivity in AD patients relative to control subjects. During the resting state following acupuncture, AD patients showed increased connectivity in most of these hippocampus related regions compared to the first resting state. In conclusion, we investigated the effect of acupuncture on AD patients by combing fMRI and traditional acupuncture. Our fMRI study confirmed that acupuncture at Tai chong and He gu can enhance the hippocampal connectivity in AD patients.

## Introduction

Alzheimer disease (AD) is the most common cause of dementia, and there is currently no effective therapy for the disease. Acupuncture has shown promise in treating AD patients by mobilizing the neurophysiological system to modulate cognitive function [1]. However, the neural mechanism underlying the effects of acupuncture is still unknown.

Recently, a promising resting-state functional magnetic resonance imaging (fMRI) method has provided insight into brain activity or connectivity and can be used to assess the effects of acupuncture. Accumulating neuroimaging evidence suggests that acupuncture can modulate resting state brain activity or connectivity [2]–[6]. However, the majority of these studies have focused on healthy subjects. Only two papers have investigated acupuncture’s effects on AD patients. One study found that acupuncture activates several temporal lobe and parietal lobe regions in AD patients [1]. Another study found that several brain regions, temporal lobe regions in particular, are activated after acupuncture in AD patients [7]. These fMRI studies suggest that acupuncture may activate regions associated with cognition, which contribute to the treatment’s specific therapeutic effect. However, these AD-related acupuncture studies did not examine connectivity changes among brain regions; instead, they explored activation of brain regions.

In order to better understand the pathophysiology of AD, it is necessary to study acupuncture’s effect on the functional connectivity of the hippocampus. Previous neuroimaging studies have shown that the hippocampus is one of the earliest pathological sites of AD and plays a crucial role in memory processes [8]–[10]. By using MRI method, many studies have demonstrated AD-related hippocampal abnormalities including atrophy [11], [12] hypometabolism [13], and decreased activity [14]. Furthermore, several fMRI studies reported markedly reduced functional connectivity in hippocampus-related memory networks in early-stage AD [15], [16] as well as mild cognitive impairment [17]–[19]. One recent resting-state fMRI study found stronger recovery of hippocampal functional connectivity after donepezil treatment in AD patients [20], which indicates there is some plasticity in hippocampal connectivity. Therefore, we selected the hippocampus as the region of interest to conduct functional connectivity analysis and to explore the effects of acupuncture.

Considering the important role of the hippocampus and acupuncture’s probable effects, we chose to observe how hippocampal activity – which is abnormal in AD patients at rest – might be affected by acupuncture. Few studies have reported changes in hippocampal connectivity in AD patients when measuring resting-state fMRI and the effects of acupuncture. Here, we hypothesized that abnormal resting-state hippocampal connectivity with vital brain regions would be enhanced in AD patients following acupuncture.

The fMRI experiment was performed after acupuncture at the acupoints Tai chong and He gu. According to traditional Chinese medicine, AD belongs to the category of dementia. When a lesion is located in the brain, acupuncture point selection should be based on inducing resuscitation and coordinating yin and yang. Huangdi Neijing says “the viscera of the disease, all take its original point”. He gu is the original point of the large intestine channel of hand yangming (LI4), and it is one of the most commonly used acupoints in the clinical therapy, playing an important role in dispelling wind and analgesia and restoring consciousness. Taichong is the original point of the liver channel of foot jueyin (Liv3), which functions to relieve the depressed liver and subdue the endogenous wind and sedatives. He gu and Tai chong are collectively named the Si Guan (four gates) point. Combined use of these two acupoints can harmonize yin and yang, regulate qi and blood, finally improve the cognitive ability of AD patients. Therefore, we choose these two acupoints.

In the present study, we sought to investigate the effects of acupuncture on hippocampal functional connectivity in AD patients compared to healthy controls. We first explored hippocampal functional connectivity during the resting state and then identified regions in AD patients showing connectivity that was significantly different compared to controls. Finally, we tested whether interregional connectivity could be modulated by acupuncture in AD patients.

## Materials and Methods

### Subjects

Twenty-eight right-handed subjects participated in this study, including 14 patients with AD and 14 healthy controls. The AD subjects were recruited from patients who had consulted in the memory clinic at Xuanwu Hospital for memory complaints. Healthy elderly controls were recruited from the local community. This study was approved by the Medical Research Ethics Committee of Xuanwu Hospital. All subjects gave their written informed consent. The details of the consent form included the study’s aim, inclusion and exclusion criteria, procedures, potential harm and benefits, medical care, privacy rights, and withdrawal process. They were informed of their right to discontinue participation at any time. All potential participants who declined to participate or otherwise did not participate were still eligible for treatment (if applicable) and were not disadvantaged in any other way for not participating in the study.

All AD patients underwent a complete physical and neurological examination, standard laboratory tests and an extensive battery of neuropsychological assessments. The diagnosis of AD fulfilled the Diagnostic and Statistical Manual of Mental Disorders 4th Edition criteria for dementia [21] and the National Institute of Neurological and Communicative Disorders and Stroke/Alzheimer Disease and Related Disorders Association (NINCDS-ADRDA) criteria for possible or probable AD [22]. The subjects were classified according to Clinical Dementia Rating (CDR) scores [23]; patients with CDRs of 1 and 2 were designated AD patients.

Healthy controls met the following criteria: a) no neurological or psychiatric disorders such as stroke, depression or epilepsy; b) no neurological deficiencies such as visual or hearing loss; c) no abnormal findings such as infarction or focal lesion in conventional brain MR imaging; d) no cognitive complaints; e) mini-mental state examination (MMSE) score of 28 or higher; and f) CDR score of 0.

Participants with contraindications for MRI such as pacemakers, cardiac defibrillators, implanted material with electric or magnetic systems, vascular clips or mechanical heart valves, cochlear implants or claustrophobia were excluded. In addition, patients with a history of stroke, psychiatric disease, drug abuse, severe hypertension, systematic diseases and intellectual disability were excluded.

### Data Acquisition

MRI data acquisition was performed using a SIEMENS verio 3-Tesla scanner (Siemens, Erlangen, Germany). The subjects were instructed to remain still, keep their eyes closed and think of nothing in particular. fMRI was acquired axially using echo-planar imaging (EPI) [repetition time (TR)/echo time (TE)/flip angle (FA)/field of view (FOV) = 2000 ms/40 ms/90°/24 cm, image matrix = 64×64, slice number = 33, thickness = 3 mm, gap = 1 mm and bandwidth = 2232 Hz/pixel]. In addition, 3D T1-weighted magnetization-prepared rapid gradient echo (MPRAGE) sagittal images were obtained (TR/TE/inversion time (TI)/FA = 1900 ms/2.2 ms/900 ms/9°, image matrix = 256×256, slice number = 176 and thickness = 1 mm).

**Figure 1:**
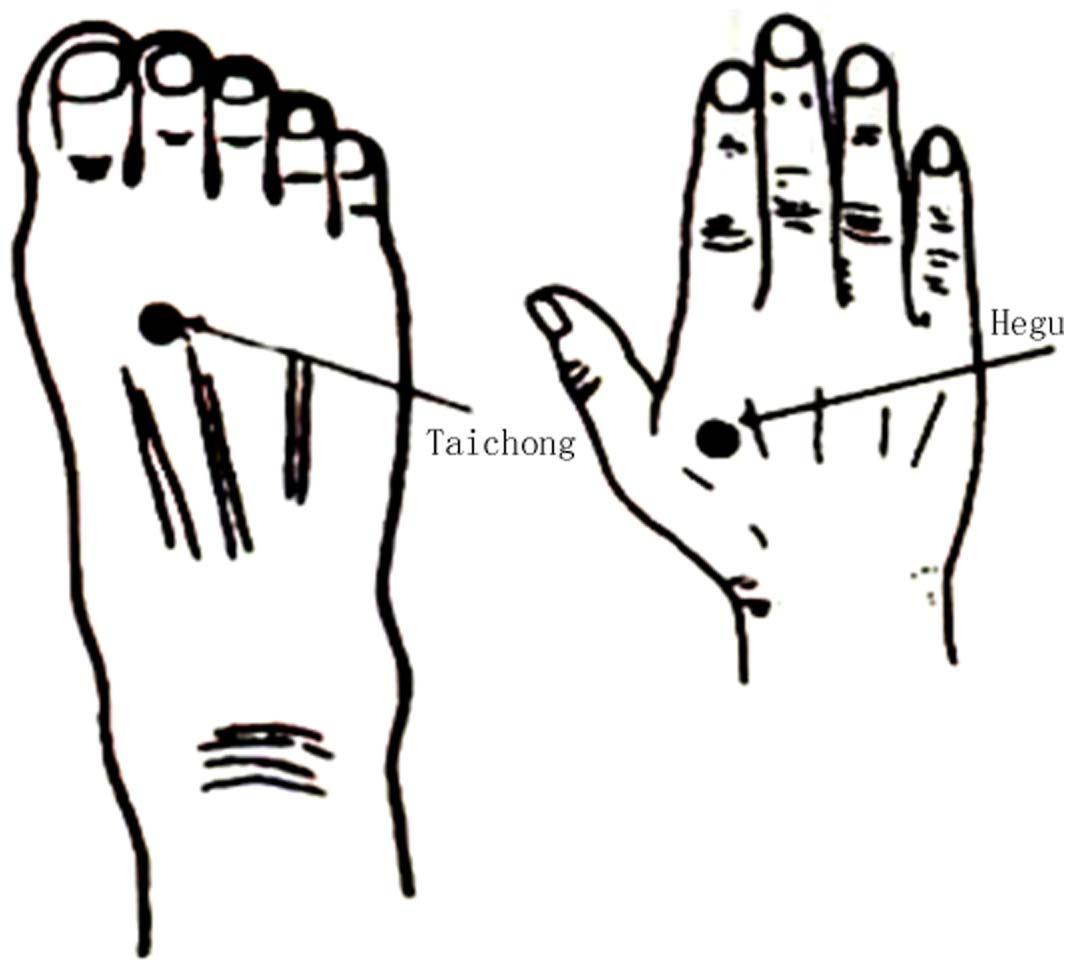
Location of acupoints used in the experiment. The left acupoint represent Taichong, which is located in the dorsal foot, the first and second metatarsal cavity before integration. The right acupoint represent Hegu, which is located in the dorsal hand, between the first and second metacarpal and the midpoint of the second metacarpal radial side.

Our study used a single block experimental design. We first acquired baseline resting state data during the initial 3 minutes; we then performed acupuncture stimulation for the following 3 minutes. A silver needle that was 0.30 mm in diameter and 25 mm long was inserted and twirled at four acupoints of the human body, which were Tai chong on the dorsum of the left and right feet and He gu on the dorsum of the left and right hands. We acquired fMRI for another 10 minutes after the needle was withdrawn. The location of Tai chong and He gu see the Figure 1.

### Imaging Preprocess

Unless otherwise stated, all analyses were conducted using a statistical parametric mapping software package (SPM5, http://www.fil.ion.ucl.ac.uk/spm). The first 10 volumes of functional images were discarded to allow the signal to reach equilibrium and to let participants adapt to the scanning noise. The remaining 229 fMRI images were first corrected for within-scan acquisition time differences between slices and then realigned to the first volume to correct for inter-scan head motion. No participant had head motion of more than 1.5 mm displacement in any of the x, y, or z directions or 1.5° of any angular motion throughout the course of scan. The individual structural image was co-registered to the mean functional image after motion correction using a linear transformation. The transformed structural images were then segmented into gray matter (GM), white matter (WM) and cerebrospinal fluid (CSF) using a unified segmentation algorithm [24]. The motion corrected functional volumes were spatially normalized to the Montreal Neurological Institute (MNI) space and re-sampled to 3 mm isotropic voxels using the normalization parameters estimated during unified segmentation. Subsequently, the functional images were spatially smoothed with a Gaussian kernel of 4×4×4 mm^3^ full width at half maximum (FWHM) to decrease spatial noise. Following this, temporal filtering (0.01 Hz<f <0.08 Hz) was applied to the time series of each voxel to reduce the effect of low-frequency drifts and high-frequency noise [25], [26] using the Resting-State fMRI Data Analysis Toolkit (http://resting-fmri.sourceforge.net). To further reduce the effects of confounding factors, we also regressed out the following confounding sources [27]: (1) six motion parameters, (2) linear drift, (3) white matter signal and (4) CSF signal.

### Region of Interest Definition

Bilateral hippocampus region of interests (ROIs) were generated using the free software WFU_PickAtlas Tool Version 2.4 (http://www.ansir.wfubmc.edu) [28], which has been used in previous studies [18], [29]. For each seed region, the blood oxygenation level dependent (BOLD) time series of the voxels within the seed region was averaged to generate the reference time series.

### Functional Connectivity Analysis

For each subject and each seed region (bilateral hippocampus), a correlation map was produced by computing the correlation coefficients between the reference time series and the time series from all the other brain voxels. Correlation coefficients were then converted to z values using Fisher r-to-z transformation to improve normality [30].

### Statistical Analysis

The individual z value was entered into a random effect one sample t-test in a voxel-wise manner to determine brain regions showing significant connectivity with the left and right hippocampus within each group under a combined threshold of P<0.01 and cluster size = 405 mm^3^. This yielded a corrected threshold of P<0.001, determined by Monte Carlo simulation using the AlphaSim program with the following parameters: FWHM = 4 mm, within the GM mask (http://afni.nimh.nih.gov/pub/dist/doc/manual/AlphaSim.pdf). This procedure produced significant hippocampal functional connectivity z-statistic maps for the two groups (AD patients and controls). We then made masks showing the bilateral hippocampus by combining the corresponding two z-statistic maps (i.e., AD patients and controls) and then used this mask for analyzing the corresponding group differences.

The z values were also entered into a random effect two-sample t-test to identify regions showing significant differences between AD patients and controls in connectivity with the bilateral hippocampus. Voxels that passed a corrected threshold of P<0.01 (group differences between AD patients and healthy controls: single voxel threshold of P<0.01 and cluster size 270 mm^3^, using the AlphaSim program with parameters FWHM = 4 mm with mask) indicated a significant difference between the two groups.

Based on the group differences (between AD and healthy controls) of the baseline resting state, we would define functional ROIs according to activated clusters. Our aim was to determine if the effect of acupuncture can ameliorate the functional pathways. First, we extracted the z values for the pre-acupuncture and post-acupuncture stage for both AD and controls. Then, an independent-sample t-test was run for each of the ROIs (pre-acupuncture versus post-acupuncture).

## Results

### Clinical Data and Neuropsychological Test

Demographic characteristics and neuropsychological scores are shown in Table 1. There were no significant differences between the two groups in gender, age, or years of education, but the neuropsychological test scores, such as the MMSE and auditory verbal learning test (AVLT) scores, were significantly different (P<0.01) between the two groups.

**Table 1:** Characteristics of the AD patients and Normal controls.

Characteristics	AD	NOR	*P* value
N (M/F)	14(4/10)	14(6/8)	–
Age, years	66.92±8.91	66.07±5.78	0.86*
Education, years	10.07±3.38	11.00±4.52	0.61*
MMSE	15.92±4.32	28.00±1.41	<0.01*
AVLT(immediate)	11.35±3.95	26.86±5.24	<0.01*
AVLT(delayed)	2.64±1.59	11.07±2.76	<0.01*
AVLT(recognition)	3.35±1.55	12.71±2.09	<0.01*
CDR	1–2	0	–

MMSE, Mini-Mental State Examination; Plus-minus values are means ± S.D. AVLT, Auditory verbal learning test; immediate, immediate recall of learning verbal; delayed; delayed recall of learning verbal; recognition, recognition of learning verbal; CDR, clinical dementia rate. *The *P* values were obtained by one-way analysis of variance tests.

### Comparisons of the Hippocampal Connectivity between AD Patients and Healthy Controls in the Resting State

**Figure 2:**
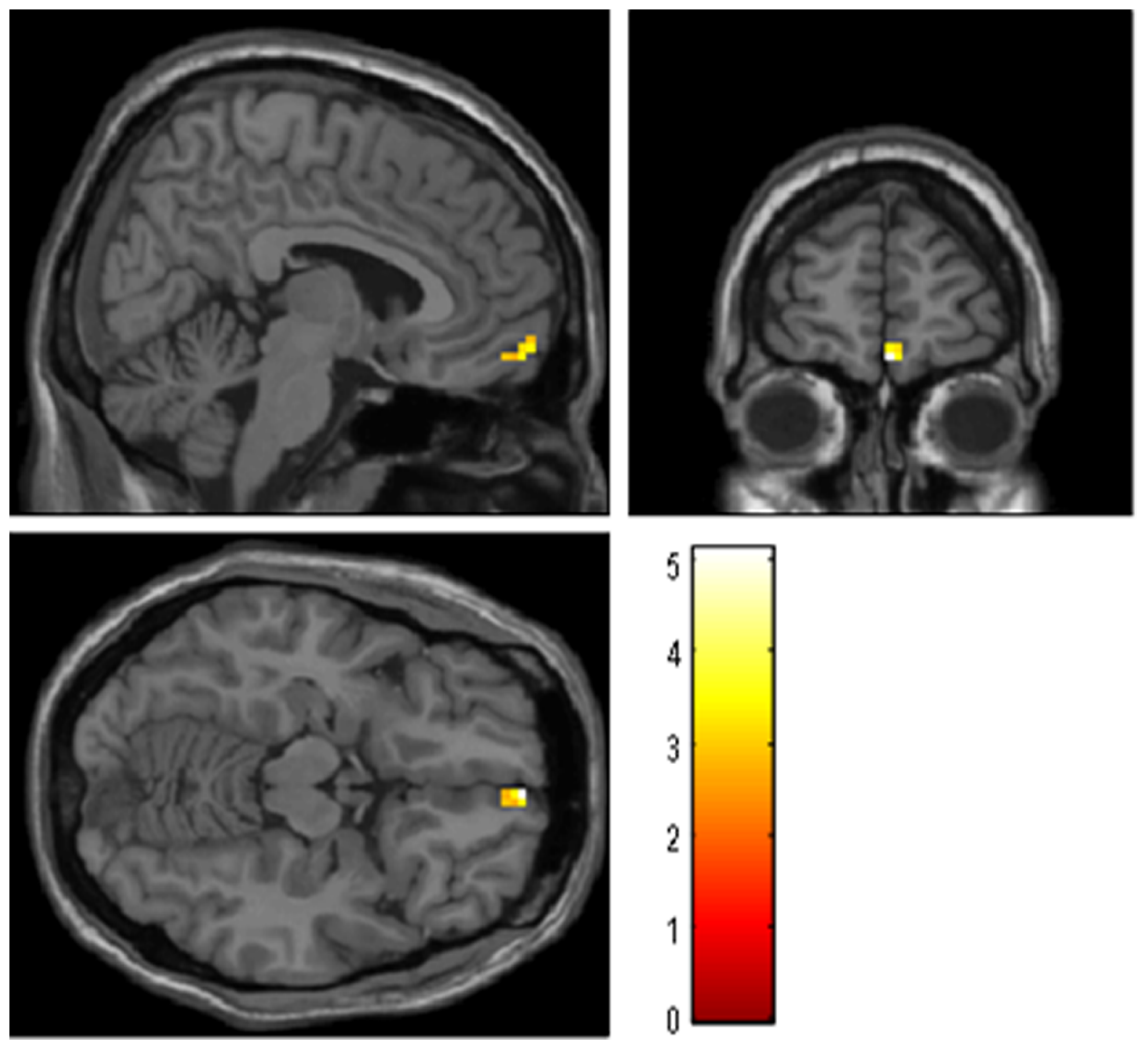
Brain regions showing decreased connectivity to left hippocampus in AD group comparing to control group. Left in picture is left in the brain. The color scale represents t values. Warm color represents decreased connectivity.

**Figure 3:**
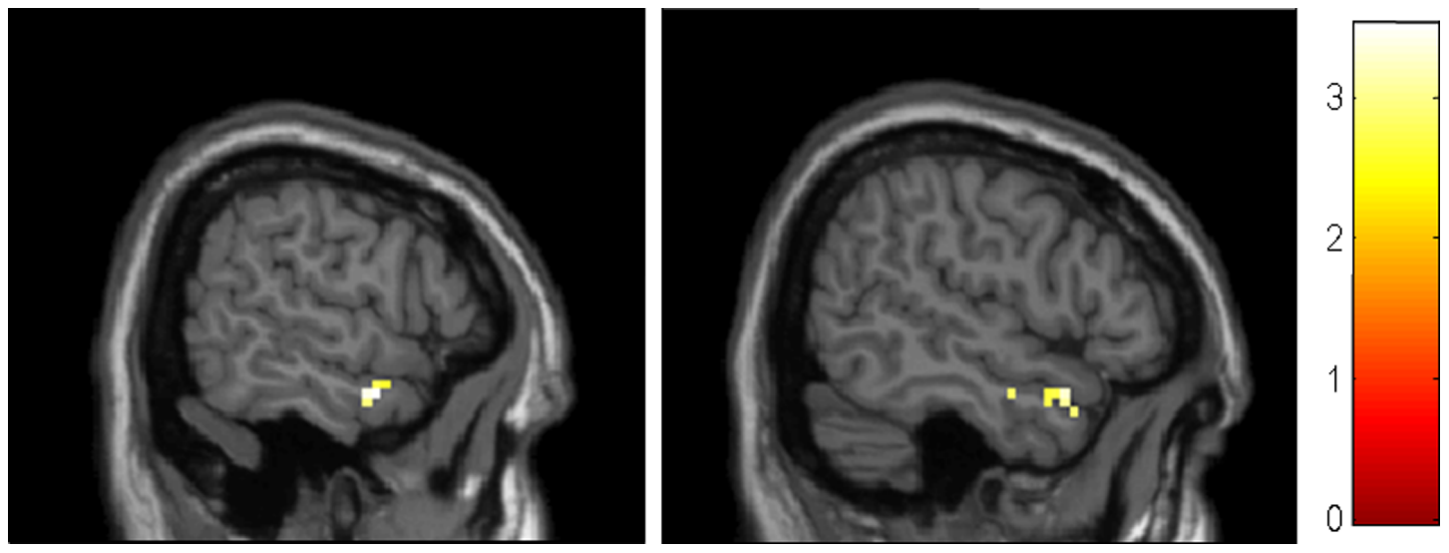
Brain regions showing decreased connectivity to right hippocampus in AD group comparing to control group. Left in picture is left in the brain. The color scale represents t values. Warm color represents decreased connectivity.

We compared the AD vs. control group resting state data to find the regions in AD patients that showed abnormal functional connectivity (shown in Table 2, Figures 2 and 3). When comparing left hippocampus connectivity between the AD and control groups, the right medial prefrontal cortex (MPFC) showed significantly decreased connectivity with the left hippocampus in the AD group. There were no regions showing increased connectivity with the left hippocampus in the AD group.

**Table 2:** Regions showing decreased and increased hippocampal connectivity in AD subjects during resting state.

Brain Regions	BA	Cluster	Coordinates (MNI)			T-score
		size	x	y	z	
**Left hippocampus**						
NC>AD						
R MPFC	11	12	3	60	−5	5.12
AD>NC						
None						
**Right hippocampus**						
NC>AD						
R ITG	21	12	60	0	−21	3.51
	20		51	−6	−21	3.06
R STG	38	13	54	12	−21	3.22
AD>NC						
None						

*P*<0.01, uncorrected, extent threshold = 10. BA Broadman area. MNI, Montreal Neurological Institute; x, y, z, coordinates of primary peak locations in the MNI space. T value represents differences of hippocampal connectivity in AD and normal controls. MPFC, medial prefrontal cortex; MTG, middle temporal gyrus; ITG, inferior temporal gyrus; STG, superior temporal gyrus.

When comparing the right hippocampal connectivity between the AD and control groups, there was significantly decreased hippocampal connectivity with the right superior temporal gyrus (STG) and the inferior temporal gyrus (ITG) in the AD group. No regions showed increased connectivity with the right hippocampus in the AD group.

### Comparisons of the ROI Functional Connectivity between Post-acupuncture and Pre-acupuncture in the AD Patients

**Figure 4:**
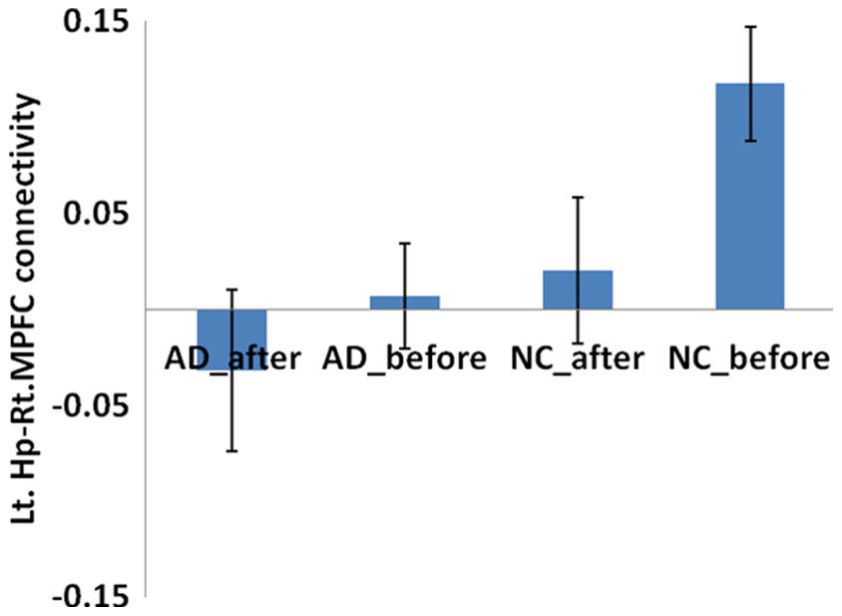
Comparison of connectivity of left hippocampus-right MPFC between pre and post acupuncture among AD patients and controls. There is a significant decreased connectivity following acupuncture for healthy controls (t = 1.942, p = 0.033). However, the AD patients showed negative connectivity after acupuncture, the different is not significant for the AD patients (t = 0.774, p = 0.225),

There was a significant decrease in the connectivity between the left hippocampus and right MPFC in healthy controls following acupuncture (t = 1.942, p = 0.033). The AD patients showed decreased connectivity between the left hippocampus and right MPFC, but the difference was not significant in the AD patients (t = 0.774, p = 0.225). For detailed data on the regions, see Figure 4.

**Figure 5:**
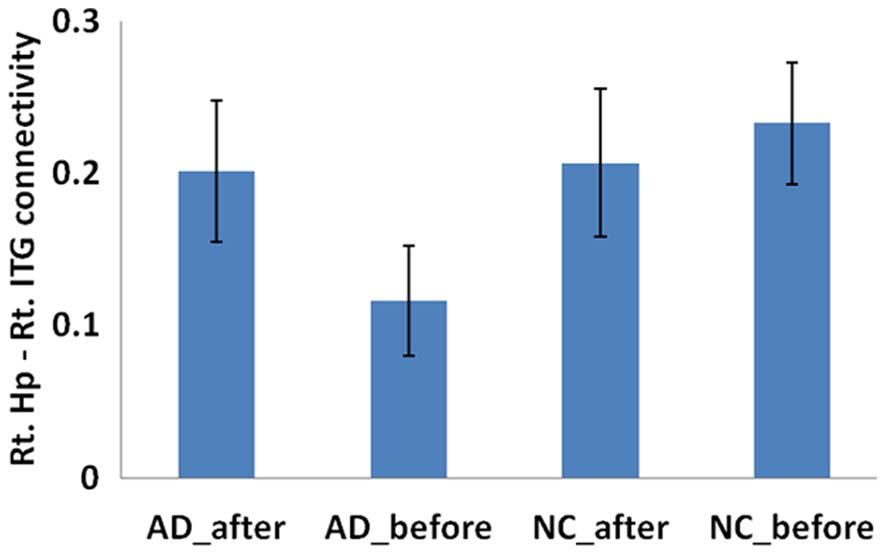
Comparison of connectivity of right hippocampus and right ITG between pre and post acupuncture among AD patients and controls. The acupuncture induced increased functional connectivity for the AD patients (t = 1.61, p = 0.056). There was no significant difference for healthy controls (t = 0.403,p = 0.345).

**Figure 6:**
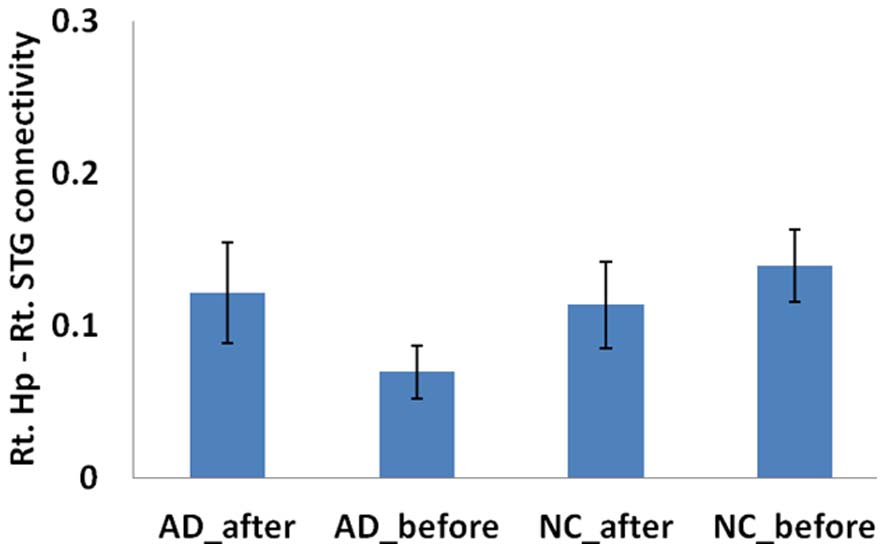
Comparison of connectivity of right hippocampus and right STG between pre and post acupuncture among AD patients and controls. The acupuncture induced increased functional connectivity for the AD patients (t = 1.458, p = 0.08). There was no significant difference for healthy controls (t = 0.675, p = 0.254).

Connectivities between the right hippocampus and right ITG and STG were lower following acupuncture in the control group, but the differences were not significant (right ITG: t = 0.403, p = 0.345; right STG: t = 0.675, p = 0.254); the AD patients showed higher connectivity after acupuncture (right ITG: t = 1.61, p = 0.056; right STG: t = 1.458, p = 0.08) and these results did not reach but approach significance. For the details of the regions, see Figures 5 and 6.

### Comparisons of the Whole Brain Hippocampal Connectivity Post-acupuncture and Pre-acupuncture in AD Patients

**Figure 7:**
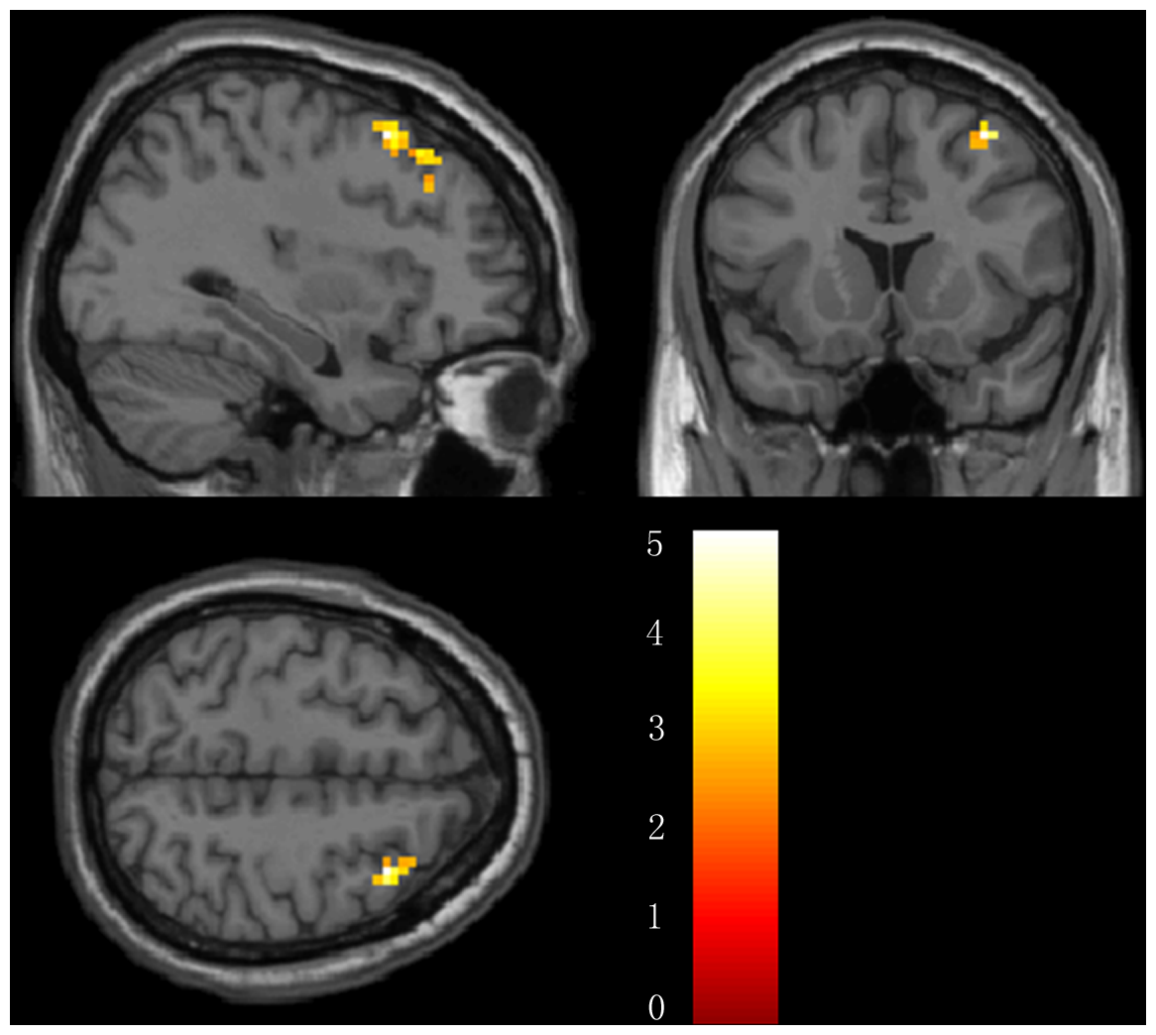
Brain regions showing altered connectivity to left or right hippocampus in AD group after acupuncture comparing to before acupuncture. The right MFG showed increased connectivity to left hippocampus in AD patients after acupuncture. Left in picture is left in the brain. The color scale represents t values.

We also compared the whole brain connectivity post-and pre-acupuncture in AD patients. In the AD group, we found that the right middle frontal lobe (MFG) showed significantly higher connectivity with the left hippocampus following acupuncture. There were no other regions showing altered connectivity with the left or right hippocampus in the AD group. For the details of the regions, see Table 3 and Figures 7.

**Table 3:** Regions showing decreased and increased hippocampal connectivity in AD subjects after acupuncture comparing to before acupuncture.

Brain Regions	BA	Cluster	Coordinates (MNI)				T-score
		size	x	y	z		
**Left hippocampus**							
AD after>AD before							
R MFG	8	25	39	30		45	4.04
R MFG	9		39	30		36	3.11
AD before>AD after							
None							
**Right hippocampus**							
None							

*P*<0.01 uncorrected, extent threshold = 20. BA, Broadman area. MNI, Montreal Neurological Institute; x, y, z, coordinates of primary peak locations in the MNI space. T value represents differences of hippocampal connectivity in AD and normal controls. MFG, middle frontal gyrus.

### Comparisons of the Whole Brain Hippocampal Connectivity Post-acupuncture and Pre-acupuncture in Normal Control Subjects

**Figure 8:**
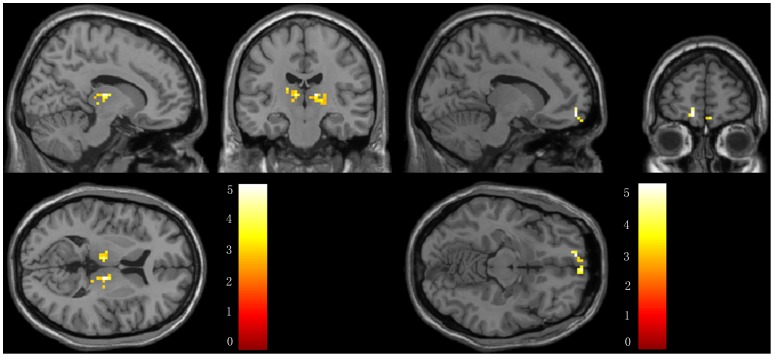
Brain regions showing altered connectivity to left hippocampus in control group after acupuncture comparing to before acupuncture. The thalamus showed increased connectivity while the MPFC showed decreased connectivity to left hippocampus in controls after acupuncture. Left in picture is left in the brain. The color scale represents t values.

**Figure 9:**
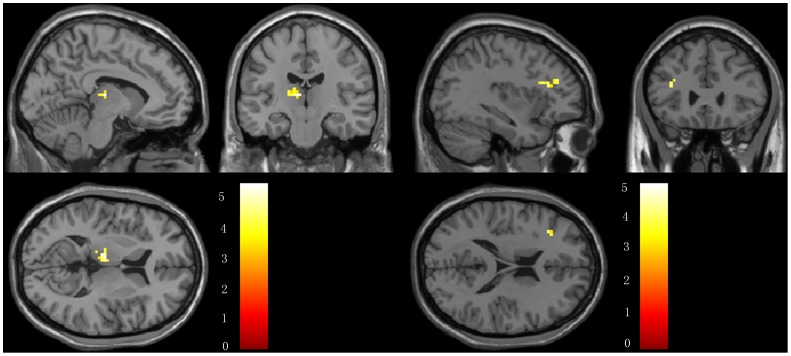
Brain regions showing altered connectivity to right hippocampus in control group after acupuncture comparing to before acupuncture. The left thalamus, MFG and insula showed increased connectivity to right hippocampus in controls after acupuncture. Left in picture is left in the brain. The color scale represents t values.

For reference, we also made a comparison of the post- and pre-acupuncture hippocampal connectivity in the normal control subjects. We found that the bilateral thalamus showed significantly higher connectivity with the left hippocampus, while the MPFC showed significantly lower connectivity with the left hippocampus. In addition, the left thalamus, MFG and insula showed significantly higher connectivity with the right hippocampus following acupuncture in the control group. For the details of the regions, see Table 4 and Figures 8 and 9.

**Table 4:** Regions showing decreased and increased hippocampal connectivity in normal controls after acupuncture comparing to before acupuncture.

Brain Regions	BA	Cluster	Coordinates(MNI)				T-score
		size	x	y		z	
**Left hippocampus**							
Controls after>Controls before							
R Thalamus	–	56	12		−18	9	4.69
R Thalamus	–		6		−9	3	4.62
R Thalamus	–		15		−24	3	4.33
L Thalamus	–	23	−18		−21	15	4.23
L Thalamus	–		−9		−18	9	4.14
Controls before>Controls after							
L MPFC	11	27	−12		57	−12	4.33
R MPFC	11		3		63	−12	3.76
**Right hippocampus**							
Controls after>Controls before							
L Thalamus	–	23	−9		−18	9	4.20
L Thalamus	–		−18		−24	12	3.71
L MFG	46	25	−39		36	24	3.47
L Insula	13		−36		21	21	3.42

*P*<0.01 uncorrected, extent threshold = 20. BA, Broadman area. MNI, Montreal Neurological Institute; x, y, z, coordinates of primary peak locations in the MNI space. T value represents differences of hippocampal connectivity in normal controls between post-acupuncture and pre-acupuncture. MPFC, medial prefrontal cortex; MFG, middle frontal gyrus.

In order to see if there is any enhancement in the acupuncture group comparing with the non acupuncture group among patients with the same illness, we selected another non acupuncture AD group as reference to compare the differences of hippocampal connectivity. To keep the structure of the paper, we put the results into the supplementary material. As the results, we found several regions such as the bilateral medial temporal gyrus (MTG), left ITG and fusiform gyrus (FG) showed increased hippocampal connectivity when comparing acupuncture AD group to the non-acupuncture AD group. The results are consistent with our other results. The details see Table S1, S2 and Figure S1, S2 in File S1.

## Discussion

Recently, several researchers have begun to pay attention to the sustained effect of acupuncture and its influence on the resting brain. For the first time, we used fMRI hippocampal connectivity analysis to explore the sustained effect of acupuncture on AD patients. The following are the two main findings of the present study: first, multiple regions show disrupted connectivity in the hippocampus in AD patients. Most of these regions are involved in the hippocampal–cortical memory system. Second, hippocampal connectivity with the frontal and lateral temporal regions in AD patients showed enhancement after acupuncture.

During the resting state, we found abnormal hippocampal functional connectivity in the AD patients relative to controls. The right MPFC showed decreased connectivity with the left hippocampus in the AD patients. The MPFC is considered an important component of human default-mode networks (DMN) [25], [31]–[32]. In previous resting fMRI studies, it has been suggested that a disrupted connection between the MPFC and hippocampus may represent decreased activity of the DMN and contribute to memory impairment in AD patients [15]–[16], [33]. In addition, the right temporal regions (STG and ITG) showed decreased connectivity with the right hippocampus in AD patients. According to previous studies, the lateral temporal cortex, including the STG and ITG, is consistently involved with the DMN, though connectivity is less robust [31]. The STG and ITG regions are important components of the DMN, and AD patients present with amyloid deposits, hypometabolism and atrophy in those regions of the brain [12]. Using resting-state fMRI analysis, disrupted connections between the right hippocampus and temporal regions have also been reported in early-stage AD patients [15]–[16], [18]. Together, our results are largely consistent with those reported by previous studies.

When comparing connectivity of the ROIs post and pre-acupuncture, we found enhanced connectivity between the right temporal regions (STG and ITG) and the hippocampus following acupuncture. Currently, very little is known about how acupuncture affects functional brain connectivity in patients with mild AD. Previous studies suggest that acupuncture can modulate resting state brain connectivity [2]–[6], and we speculate that enhanced connectivity between the right temporal regions and hippocampus may relate to the specific regulatory effect of acupuncture. Due to the cognitive impairment associated with AD, acupuncture on specific acupoints can modulate the cerebral blood flow and strengthen the hippocampal connectivity in AD patients. In a recent acupuncture study related to mild cognitive impairment (MCI), increased hippocampal connectivity was detected after acupuncture on acupoint KI3 [34], which was consistent with our study. Our study provides new evidence that acupuncture has a striking, sustained effect on AD patients.

Although no significant differences were found post-acupuncture in the connectivity between the right MPFC and left hippocampus using ROI analysis, whole brain analysis revealed enhanced connectivity between the regions in AD patients. Previous experiments using task or resting-state fMRI have reported increased activity or connectivity in the frontal region of AD patients compared to healthy controls [15], [35]–[37]. A recent resting fMRI study showed enhanced functional connectivity within the frontal cortex of early-stage MCI patients [38]. These previous studies suggest that AD patients can use additional neural resources to preserve and compensate for losses in memory function attributable to the degenerative effects of the disease. In our study, due to the severe pathological changes in AD patients, the AD group did not have any regions with increased connectivity with the hippocampus during the initial resting state. However, we noticed increased connectivity between the MFG and hippocampus after acupuncture, which suggests that acupuncture may exert modulatory effects on hippocampal connectivity. We speculate that acupuncture activates the compensatory processes in AD patients and strengthens cooperation between brain regions, which is compatible with the theory of dynamic functional reorganization [35], [39].

As we all know, hippocampal atrophy correlates significantly with cognitive decline in patients with AD as well as MCI. Thus, assessing and monitoring hippocampal function are very useful for evaluation of AD. Recently, resting state fMRI provided a new promising method for exploring hippocampal function by investigating coherence in the fMRI signal between hippocampus and all other brain regions. In a previous longitudinal study of our group, we explored the correlation between strength of hippocampal connectivity and neuropsychological data to study whether changes in hippocampal connectivity could reflect the cognitive decline in MCI, the early stage of AD. As the result, we found the strength of the ITG-hippocampus connectivity showed significant positive correlation with MMSE scores [18]. This implied that the ITG- hippocampus decreased connectivity may contribute to the cognitive decline in the MCI patients. In another longitudinal MCI study from other group, they also found functional connectivity of hippocampus is associated with MFG, lateral temporal cortex, posterior cingulate cortex and other regions, some of them showed positive correlation with episode memory scores [19]. In addition, some researchers found disruption of hippocampal functional connectivity in AD patients indicating of the cognitive impairment [15], [16].Collectively, based on the previous study, we speculated that enhanced hippocampal connectivity, which is induced by acupuncture in the current study, can enhance the information flow and result in improvement of cognitive function in AD patients.

In the current study, we noticed asymmetry between the left and right brain regions during the resting state. The right MPFC, right STG and ITG showed decreased connectivity with the hippocampus in the AD group; after acupuncture, we found enhanced connectivity between the right MFG and left hippocampus in AD patients, which is interesting. We reviewed the literature, and several MRI studies report rightward asymmetry in the volume of the hippocampus in the normal adults, [40], [41] and this anatomically rightward asymmetry was diminished in AD patients [42], [43]. Furthermore, in a recent study of AD patients, the authors reported that hippocampal connectivity in normal controls presents with rightward asymmetry, which is diminished in AD patients [15]. In the current study, we found decreased connectivity between the right MPFC, STG and ITG and the hippocampus in AD patients during the resting state, which is consistent with the previous studies. Based on the relationship between the structure and function, we attribute the rightward asymmetry of the hippocampal connectivity to the rightward asymmetry of the hippocampal structure.

There are several limitations of this study. First, an obstacle to imaging the effects of acupuncture is isolating the brain activity related to sensory stimulation from the brain activity associated with any potential therapeutic effects. Even after the needle is withdrawn, the subjects might be influenced by sensory stimulation. Second, the current study would benefit from including a control state that could be compared with real-needle acupuncture, such as sham acupuncture. Third, we didn’t show cognitive performances correlations with the functional connectivity and the changes induced by the acupuncture treatments. a longitudinal design will be necessary to determine the impacts of acupuncture on hippocampal connectivity and cognitive performances. In the future, we will trace these subjects using different time points and explore potential hippocampal connectivity changes and their influence on cognitive function in AD patients after acupuncture.

In conclusion, our results revealed the acupuncture at Tai chong and He gu can enhance the hippocampal connectivity in the AD patients. It may provide deep understanding of the therapeutic effect of acupuncture and open a new avenue for the treatment of AD in the future.

## Supporting Information

File S1 **Supporting Information. Figure S1, Brain regions showing increased connectivity to left hippocampus in acupuncture AD1 group comparing to non-acupuncture AD2 group.** These regions include left MTG and FG. **Figure S2, Brain regions showing increased connectivity to right hippocampus in acupuncture AD1 group comparing to non-acupuncture AD2 group.** These regions include left FG, ITG and the right MTG. **Table S1, Characteristics of the acupuncture AD1 patients and non-acupuncture AD2 group. Table S2, Regions showing increased hippocampal connectivity in AD1 group after acupuncture comparing to another non-acupuncture AD2 group.**
(DOC)
