# Supplementary material for: Acupuncture Modulates Resting State Hippocampal Functional Connectivity in Alzheimer Disease
Source: PLoS One. 2014 Mar 6;9(3):e91160. doi: 10.1371/journal.pone.0091160 (PMC3946345; doi:10.1371/journal.pone.0091160)
Supplement: File S1 — Supporting Information. Figure S1, Brain regions showing increased connectivity to left hippocampus in acupuncture AD1 group comparing to non-acupuncture AD2 group. These regions include left MTG and FG. Figure S2, Brain regions showing increased connectivity to right hippocampus in acupuncture AD1 group comparing to non-acupuncture AD2 group. These regions include left FG, ITG and the right MTG. Table S1, Characteristics of the acupuncture AD1 patients and non-acupuncture AD2 group. Table S2, Regions showing increased hippocampal connectivity in AD1 group after acupuncture comparing to another non-acupuncture AD2 group. (DOC) [file pone.0091160.s001.doc]

**Table S1 Characteristics of the acupuncture AD1 patients and non-acupuncture AD2 group**

| **Characteristics** | AD1 | AD2 | *P* |
| --- | --- | --- | --- |
| N (M/F) | 14(4/10) | 14(6/8) | - |
| Age, years | 66.92±8.91 | 67.05±6.78 | 0.86 |
| Education, years | 10.07±3.38 | 11.00±3.42 | 0.65 |
| MMSE | 15.92±4.32 | 17.00±2.41 | 0.81 |
| CDR | 1-2 | 1-2 | - |

MMSE, Mini-Mental State Examination; Plus-minus values are means ± S.D. CDR, clinical dementia rate. *The *P* values were obtained by two-sample two-tailed t test. AD1, acupuncture AD group; AD2, non acupuncture AD group.

**Table S2. Regions showing increased hippocampal connectivity in AD1 group after acupuncture comparing to another non-acupuncture AD2 group**

| **Brain Regions** | **BA** | | **Cluster**  **size** | **Coordinates (MNI)** | | | **T-score** |
| --- | --- | --- | --- | --- | --- | --- | --- |
|  |  |  |  | **x y z** | | |  |
| **Left hippocampus** |  | |  |  |  |  |  |
| AD1 > AD 2 |  | |  |  |  |  |  |
| L MTG | 21 | | 17 | -48 | -18 | -18 | 2.66 |
| L FG | 20 | |  | -48 | -27 | -27 | 2.22 |
| **Right hippocampus** |  |  | |  |  |  |  |
| AD 1> AD 2 |  |  | |  |  |  |  |
| L FG | 20 | | 25 | -51 | -30 | -27 | 4.96 |
| L ITG | 20 | |  | -48 | -24 | -21 | 2.65 |
| R MTG | 21 | | 54 | 60 | -24 | -21 | 3.09 |
| R MTG | 21 | |  | 66 | -18 | -18 | 2.88 |
| R MTG | 21 | |  | 54 | -21 | -12 | 2.41 |

*P* < 0.05 uncorrected, extent threshold = 20. BA, Broadman area. MNI, Montreal Neurological Institute; x, y, z, coordinates of primary peak locations in the MNI space. AD1, acupuncture AD group; AD2, non acupuncture AD group. T value represents differences of hippocampal connectivity in between the two AD group. MTG, middle temporal gyrus. FG, Fusiform Gyrus; ITG, inferior temporal gyrus.

**
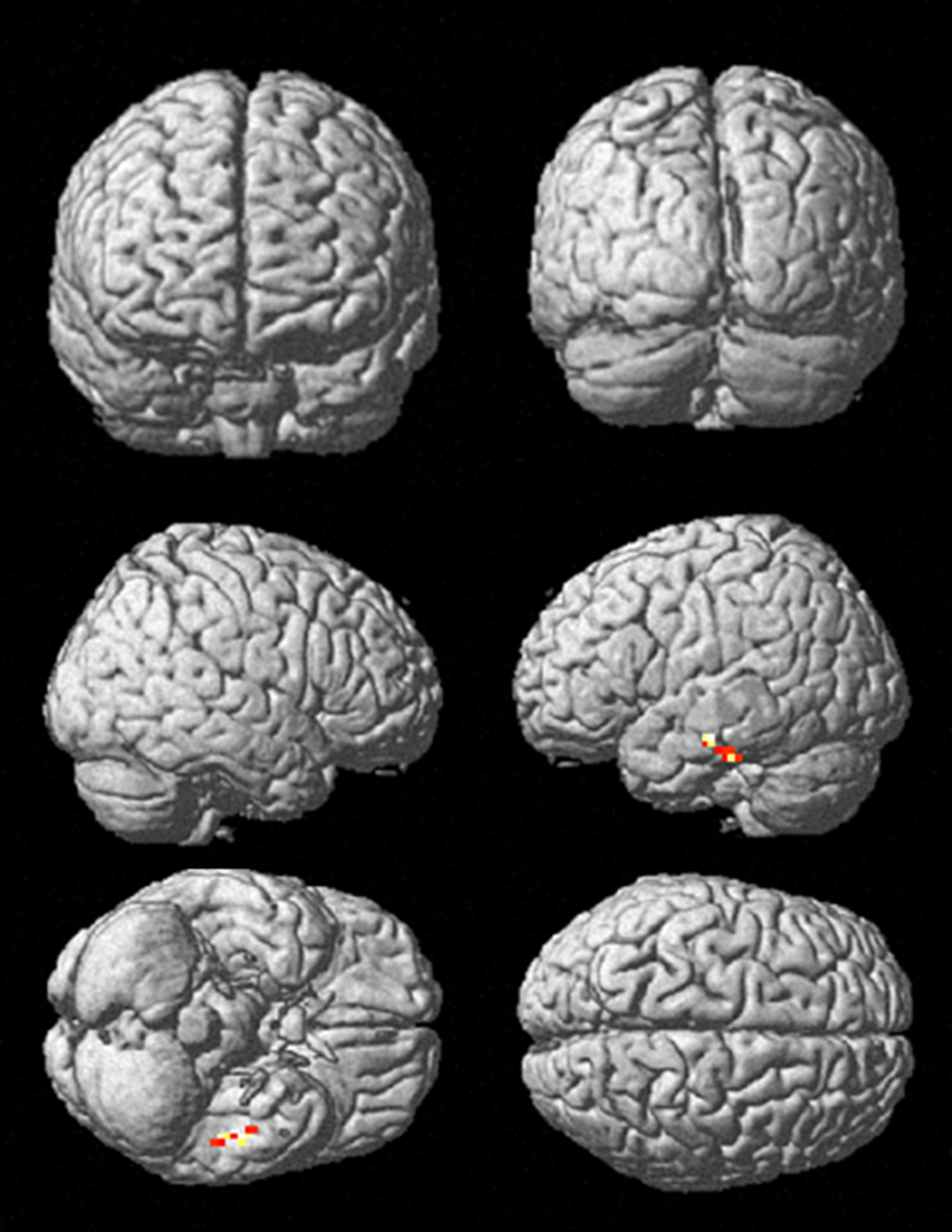
**

**Figure S1**

**
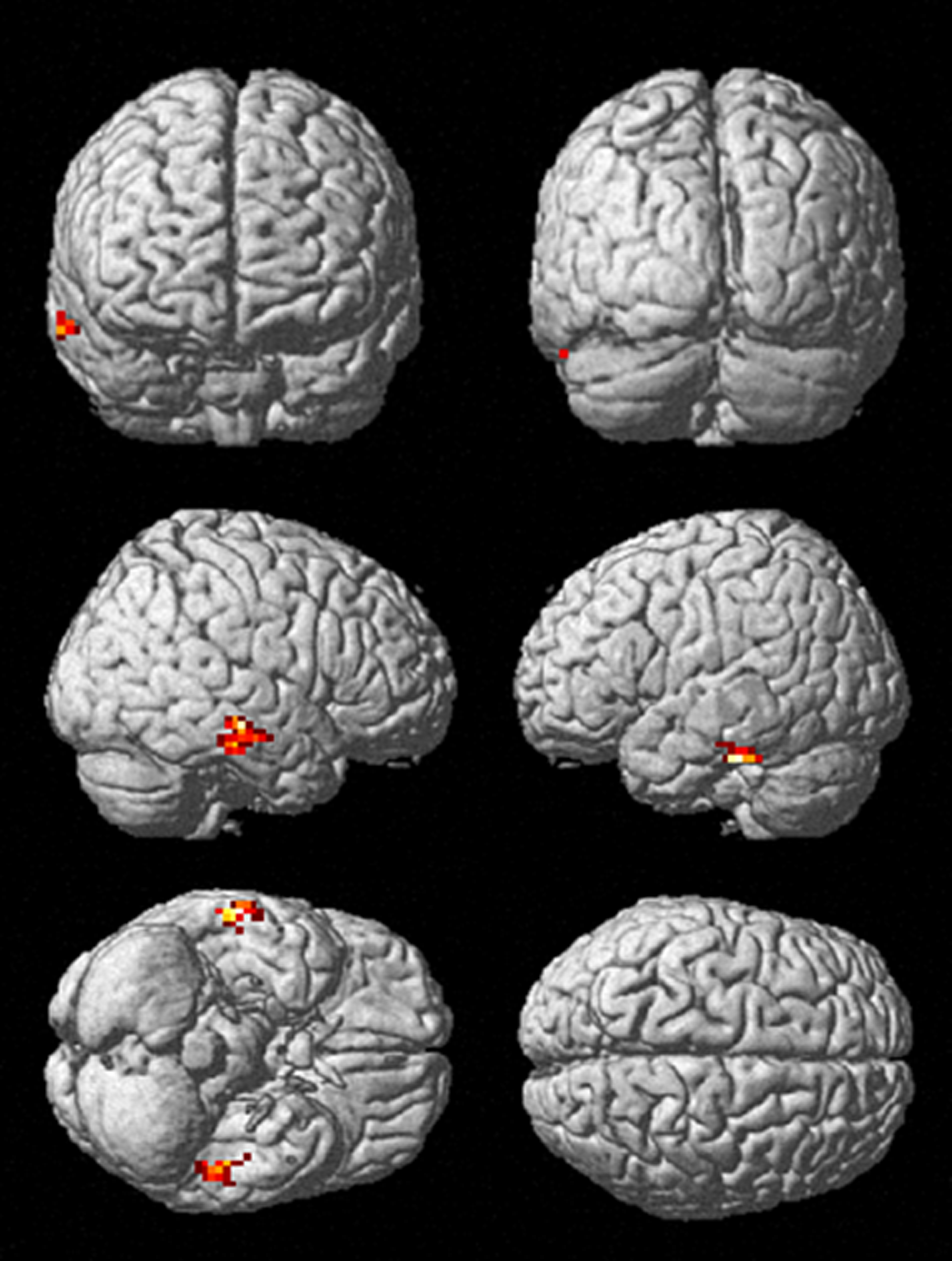
**

**Figure S2**
